# Acknowledgement of manuscript reviewers 2018

**DOI:** 10.18332/ejm/103138

**Published:** 2019-01-24

**Authors:** Victoria G. Vivilaki

**Affiliations:** 1 Department of Midwifery, University of West Attica, Aigaleo, Athens, Greece

## CONTRIBUTING REVIEWERS

The editors of ***European Journal of Midwifery*** would like to thank all our reviewers who have contributed to the journal in Volume 2 (2018).

**Cathy Ashwin**

United Kingdom

**Carrie Bonsack**

United States

**Susannah Brady**

Australia

**Jim Brigagão**

Brazil

**Irene Calvert**

New Zealand

**Megan Cooper**

Australia

**Abdolreza Daraei**

Iran

**Sarah Davies**

United Kingdom

**Rafael del Rosal**

Spain

**Ramón Escuriet**

Spain

**Gracia Fellmeth**

United Kingdom

**Sarah Fogarty**

Australia

**Hajdu Gabor**

Hungary

**Mahin Gheibizadeh**

Iran

**Enrique Gomez-Pomar**

United States

**Wendy Hall**

Canada

**Tutik Hariyati**

Indonesia

**Caroline Homer**

Australia

**Hyuk Im**

South Korea

**Lara Jansiski Motta**

Brazil

**Ingrid Jepsen**

Denmark

**Garg Kamakshi**

India

**Annika Karlstrom**

Sweden

**Bengt Kayser**

Switzerland

**Martina Koenig-Bachmann**

Austria

**Sklempe Kokic**

Croatia

**Mario Lopes**

Portugal

**Aidalina Mahmud**

Malaysia

**Daniel Martingano**

United States

**Robyn Maude**

New Zealand

**Lois McKellar**

Australia

**Polona Mivšek**

Slovenia

**Stephanie Morgan**

United States

**Tiina Murto**

Finland

**Rhona O'Connell**

Ireland

**Lucinda Roper**

Australia

**Shiri Sadeh-Sharvit**

United States

**Paula Santos**

Portugal

**Kirti Saxena**

India

**Katja Schrøder**

Denmark

**Elisabeth Severinsson**

Norway

**Susanne Simon**

Germany

**Jen Stevens**

United States

**Seyed Saeed Tabatabaee**

Iran

**Melania Tudose**

Romania

**Deborah Turnbull**

Australia

**Joeri Vermeulen**

Belgium

**Victoria Vivilaki**

Greece

**Therese Wiegers**

Netherlands

**Xianglong Xu**

China

